# Polydatin-curcumin formulation alleviates CTD-ILD-like lung injury in mice via GABBR/PI3K/AKT/TGF-β pathway 

**DOI:** 10.3389/fphar.2025.1573525

**Published:** 2025-06-05

**Authors:** Zhengju Zhang, Yao Zhang, Qingyi Lu, Yanan Wang, Guodong Li, Guoju Jin, Weiguo Ma, Yuyue Liu, Lei Yang, Hui Liu, Honghong Zhang, Wen Gu, Xinqi Deng, Chunguo Wang, Fengxian Meng

**Affiliations:** ^1^ Beijing University of Chinese Medicine, Beijing, China; ^2^ Capital Medical University, Beijing, China; ^3^ Changping District Hospital of Integrated Traditional Chinese and Western Medicine, Beijing, China; ^4^ Shunyi Hospital, Beijing Traditional Chinese Medicine Hospital, Beijing, China; ^5^ Institute Of Chinese Materia Medica China Academy of Chinese Medical Sciences, Beijing, China

**Keywords:** CTD-ILD, polydatin, curcumin, GABBR, PI3K/AKT/TGF-β

## Abstract

Connective tissue disease-associated interstitial lung disease (CTD-ILD) is a systemic autoimmune disease with high morbidity and hazard, characterized by progressive pulmonary inflammation and fibrosis. The monomer formulation of polydatin and curcumin (PD + Cur) for lung injury in CTD-ILD was optimized from Curcumae Longae Rhizoma (Curcuma Longa L.) and Polygoni Cuspidati Rhizoma Et Radix (Polygonum cuspidatum Sieb. et Zucc.). Mice with CTD-ILD-like lung injury were established by a single intratracheal drip of bleomycin. After intervening in model mice for 4 weeks, PD + Cur attenuated alveolar atrophy, fibrillar collagen formation, and thickened alveolar septa in the lung, improved serum biomarkers TOLLIP, MUC5B, KL-6, SP-D, and RCN3, and suppressed serum immunoinflammatory factors IL-6, CCL-18, and SF. The transcriptome sequencing showed that PD + Cur ameliorated CTD-ILD mainly by regulating aberrant immunoinflammation, which was further confirmed by proteomics that the PI3K/AKT/TGF-β pathway was a key pathway. Further, PD + Cur was found to affect amino acid metabolism in the serum significantly. The B-type receptor for GABA (GABBR) agonist baclofen was further found to attenuate CTD-ILD-like lung injury and modulate PI3K/AKT/TGF-β signaling. However, the inhibition of AKT, transforming growth factor beta receptor type 3 (TGFβR3), a key indicator downstream of PI3-kinase subunit p85-alpha (PI3KR1), by PD + Cur was reversed after intervention with the GABBR receptor inhibitor CGP52432. PD + Cur has an ameliorative effect on CTD-ILD-like lung injury by targeting GABBR to modulate the PI3K/AKT/TGF-β pathway.

## 1 Introduction

Connective tissue disease-associated interstitial lung disease (CTD-ILD) is a systemic autoimmune disease with immune-mediated organ dysfunction, characterized by varying degrees of immunoinflammation and fibrosis leading to lung parenchymal damage (Spagnolo et al., 2021). The incidence of CTD-ILD varies according to the type of CTD, with a high overall prevalence of 15%, leading to significant morbidity and mortality. The clinical spectrum can range from asymptomatic imaging manifestations to respiratory failure or death, placing a heavy burden on patients and the healthcare system (Tseng et al., 2023). CTD-ILD can be seen in a variety of CTDs such as systemic sclerosis (SSc), rheumatoid arthritis (RA), polymyositis (PM)/dermatomyositis (DM), Sjögren’s syndrome (SS), and systemic lupus erythematosus (SLE), which predispose to the development of interstitial pneumonitis with autoimmunity (Mulcaire-Jones et al., 2024; Storrer et al., 2024). The goal of treatment for CTD-ILD is to slow progression and maximize patient survival and quality of life. Currently, commonly used drugs include corticosteroids, mycophenolate, cyclophosphamide, *etc.*, but have side effects and even lethality (Ahmed and Handa, 2022; Storrer et al., 2024). For all these reasons, researchers are looking for alternative drugs that are effective and affordable with fewer side effects and exploring their mechanisms for treating CTD-ILD.

Polydatin and curcumin (PD + Cur) are the core pharmacological components of Curcumae Longae Rhizoma (Curcuma Longa L.) and Polygoni Cuspidati Rhizoma Et Radix (Polygonum cuspidatum Sieb. et Zucc.), respectively. Our previous studies have shown that both administered in a 1:1 dosage have a similar efficacy on ILD as the original herb (Meng and Cui, 2019). PD + Cur and PD alone were effective in ILD, reducing immunoinflammation, alveolar atrophy, and alveolar wall fibrosis in the lung tissue of ILD model mice with the advantage of few side effects (Meng and Cui, 2019; Zhang Z et al., 2024). According to studies, PD has antioxidant, anti-inflammatory, anti-apoptotic, anti-fibrotic, and other pharmacological effects (Zhang Z et al., 2024; Liu et al., 2020). Cur exhibits anti-inflammatory, immunomodulatory, antifibrotic, and antioxidant properties, and can improve respiratory disorders (Fu et al., 2021; Hanyu et al., 2023). Therefore, PD + Cur has great therapeutic potential for ILD. Clarifying the pharmacological effects of PD + Cur on ILD is conducive to developing alternative high-quality drugs for CTD-ILD, and exploring its mechanism may provide a scientific basis for its clinical application. However, no study has yet explored the mechanism of PD + Cur intervening in CTD-ILD. Moreover, studies indicate that the bleomycin–treating model can mimic the characteristics of lung damage in CTD-ILD, namely, inflammation and fibrosis, and can be used to study the mechanisms of lung injury in CTD-ILD and the improvement of drugs on it (Takei et al., 2020; Liu et al., 2022; Zhu et al., 2022). Accordingly, the present study employed a multi-omic approach, encompassing transcriptomic, proteomic, and targeted proteomic, intending to elucidate the role and mechanism of PD + Cur in the context of CTD-ILD-like lung injury induced by bleomycin, which could lay a foundation for further development of corresponding nano-targeted drugs of traditional Chinese medicine for CTD-ILD.

## 2 Materials and methods

### 2.1 Experimental procedure

All mice were acclimatized and fed for 1 week prior to formal experiments. Referring to the relevant literature for model preparation (Zhang Z et al., 2024; Zhu et al., 2022), mice were anesthetized, and 50 µL of bleomycin (1 mg mL^-1^, dissolved in PBS) was rapidly dripped into the trachea, rotating the body so that the drug diffused to the whole lung; normal controls were given 50 µL of PBS by tracheal drip in the same way as above. One week after modeling, the bleomycin–treating model was formed (Zhang Z et al., 2024; Zhu et al., 2022). Subsequently, pharmacological interventions were administered using intraperitoneal injections (i.p.) after randomization into groups.

The PD + Cur group received a mixed solution of PD and Cur (1:1) with high, medium, and low doses of 14.92, 7.46, and 3.73 mg.kg^-1^ intraperitoneally, respectively; nintedanib was administered at 17.1 mg.kg^-1^ (i.p.) to serve as a positive control; baclofen, a γ-aminobutyric acid type B receptor (GABBR) agonist, was administered at 10.0 mg kg^-1^, i. p.; CGP52432, a GABBR inhibitor, was administered intraperitoneally at 10.0 mg kg^-1^ along with the best dose of PD + Cur; the normal and model groups received the same volume of PBS, i. p. All mice were injected in a volume of 20 mL kg^-1^ every other day for 4 weeks. Six rats for each group.

### 2.2 Drugs and reagents

Bleomycin (Nippon Kayaku Seizo Co., Ltd., Japan, LOT: 420,770); Polydatin (Chengdu Herbpurify CO., Ltd., China, CAS: 27,208-80-6, RDD-H01211812016); Curcumin (Chengdu Herbpurify CO., Ltd., China, CAS: 458-37-7, RDD-J00702012004); Baclofen (Chemexpress, China, CAS: 1,134-47-0, HY-B0007); CGP52432 (Chemexpress, China, CAS: 139,667-74-6, HY-103531); Nintedanib (Beijing Hospital of Traditional Chinese Medicine, China, LOT: 203,930); ELISA kits for KL-6, SP-D, RCN3, OPN, IL-6, CCL18, and SF (eBioscience, America, ml038403V, ml037803V, ml523262V, ml001898V, ml063159V, ml037803V, ml037261 V).

### 2.3 Animals

All procedures were approved by the Ethics Committee of Beijing University of Chinese Medicine (BUCM) (No. 2023070403-3,058). SPF Male BALB/c mice (22 ± 2 g) were purchased from the Laboratory Animal Centre of Vital River Laboratories in China (Certificate No. SCXK [Jing] 2021-0,006). The animals were under controlled conditions: temperature (22°C–24°C), humidity (60%–70%), and a 12-h light/dark cycle. They were provided with *ad libitum* access to food and purified water.

### 2.4 Sample collection

Mice were anesthetized and samples were collected promptly. Serum was used for metabolomics and ELISA testing. The whole left lung was placed in 4% paraformaldehyde for H&E and Masson staining; half of the right lung was frozen in liquid nitrogen for transcriptome sequencing, and the other half in liquid nitrogen for untargeted and PRM-based targeted proteomics.

### 2.5 H&E staining

Hematoxylin and eosin (H&E) staining was performed on lung samples following the protocol reported previously (Deng et al., 2021). Sections were assessed by a masked pathologist for alveolar septal thickening and inflammatory cell infiltration. Scoring criteria for the degree of alveolar septal thickening: 0 (no thickening), 1 (≥2 times normal alveolar septum), 2 (≥4 times normal alveolar septum), 3 (≥6 times normal alveolar septum), 4 (≥8 times normal alveolar septum). The degree of interstitial inflammatory cell infiltration in the lungs was scored on a scale of 0 (no or occasional inflammatory cells seen), 1–4 (4 grades of inflammatory cell infiltration from mild to severe) (Ghosn et al., 2011; Ba et al., 2023).

### 2.6 Masson staining

Masson staining on lung samples was conducted according to the protocol provided by the manufacturer. Sections were assessed by a masked pathologist for fibroplasia of lung tissue. Semi-quantitative grading scores for fibroplasia of lung tissue based on intensity and area of staining: 0 (no or minimal fibroplasia), 1–4 (4 grades of fibroplasia from mild to severe) (Li et al., 2019).

### 2.7 ELISA analysis

ELISA technology was employed to assess plasma levels of KL-6, SP-D, RCN3, OPN, IL-6, CCL18, and SF according to the kit instructions.

### 2.8 Metabolomics analysis

Referring to previous literature (Deng et al., 2021; Naz et al., 2014), metabolites were extracted from 100 μL of serum using pure methanol. And pooled quality control (QC) samples were prepared to validate the analytical methodology. Metabolomics analysis was performed on a Thermo Scientific Vanquish UHPLC coupled to a Thermo Q Exactive Plus-Orbitrap MS system equipped with an electrospray ionization source. Waters ACQUITY UPLC HSS T3 column (2.1 × 100 mm, 1.8 μm) was equipped for data collection. The mobile phases were combined with 0.1% formic acid in water (A) and 0.1% formic acid in acetonitrile (B), and the gradient conditions were as follows: 0–1 min, 95% (A); 1–3 min, 95%–35% (A); 3–9 min, 35%–15% (A); 9–14 min, 15%–2% (A); 14–17 min, 2%–2% (A); 17–17.1 min, 2%–95% (A); 17.1–19 min, 95%–95% (A).

The operating parameters were as follows: ion source temp, 350°C; spray voltage, 4 KV for positive ionization and 3.5 KV for negative ionization; capillary temp, 320°C for positive ionization; 350 °C for negative ionization; sheath gas pressure, 35 arb; auxiliary gas pressure, 10 arb; scan modes, MS (Full Scan, m/z100-1,500) and data-dependent acquisition MS2 (resolution, 70,000 for Full Scan, 17,500 for MS2); collision energy, NEC35.

The raw data analysis was processed using Compound Discoverer 3.2 software. Principal component analysis (PCA) and orthogonal partial least squares-discriminant analysis (OPLS-DA) were carried out with SIMCA-P 14.0 software. The robustness of the OPLS-DA model was assessed through a 200-times permutation test. Differential metabolites were identified using the mzCloud database for spectral matching, in conjunction with the Metlin and HMDB databases. Metabolic pathway enrichment analysis of the identified differential components was performed using Metaboanalyst 6.0 (http://www.metaboanalyst.ca/).

### 2.9 Transcriptome sequencing (RNA-seq) analysis

Referring to previous literature (Zhang Y et al., 2024), the conventional Trizol method (Invitrogen, United States) was used to extract total RNA from the lung. Based on the NEB universal library construction approach, RNA libraries were constructed using the NEBNext^®^ Ultra™ RNA Library Prep Kit for Illumina^®^ (NEB, United States) (Parkhomchuk et al., 2009). The library was then sequenced using an Illumina Novaseq platform. Using HISAT2 software, post-quality control sequencing data were aligned with the reference genome obtained from the Ensembl database (http://ftp.ensembl.org). The feature counts function in the subread software was used to carry out the quantification of genes (Liao et al., 2014).

Differentially expressed genes (DEGs) between groups were analyzed using the DESeq2 package. Genes with a ratio ≥1.50 or Ratio ≤0.67 with a p-value <0.05 were deemed to be DEGs. The differentially expressed genes (DEGs) were subsequently annotated for Gene Ontology (GO) using the DAVID platform (https://david.ncifcrf.gov/) and enriched for pathways by Ingenuity Pathway Analysis (IPA) software.

### 2.10 Proteomics

150 μg of protein extracted from lung was enzymatically cleaved with trypsin and then desalted with Tip C18 according to prior research (Chen et al., 2023). LC-MS/MS data of proteomic fragments were captured using an Easy nano-LC II system (Thermo Fisher Scientific, Odense, Denmark) combined with an Orbitrap Fusion Lumos mass spectrometer (Thermo Fisher Scientific, Bremen, Germany). PepMap C18 75 μm × 250 mm 2 μm columns (Dr, Maisch GmbH, Germany) were used to separate peptides. The mobile phase A was a 0.1% formic acid aqueous solution, and the mobile phase B was an 80% acetonitrile solution. The separation was achieved using the gradient elution:

Mobile phases A and B were 0.1% formic acid aqueous solution and 80% acetonitrile solution, respectively, with gradient elution: 0–3 min, 6%–10% B; 3–46 min, 10%–32% B; 46–52 min, 32%–40% B; 52–55 min, 40%–90% B; 55–60 min, 95%–95% B. The mass spectrum was scanned using data-independent acquisition (DIA). Full MS method: MS resolution was 50,000, AGC was 400,000, maximum ion injection time (ITmax) was 50 m, scanning range was m/z 400–1,500, RF was 45%; DIA method: scanning resolution was 15,000, scanning range was automatic, ITmax was 22 m, AGC was 100,000, Stepped Normalized Collisional Energy was 32% by HCD cleavage. Data were processed with XCalibur (version 4.2; Thermo Fisher Scientific).

The original mass spectrometry data were searched using DIA-NM1.8.1 software and Mouse 10,090_Proteome_UP000000589 (2023.11.20) canonical sequences as search databases. The specific parameters were as follows: whole trypsin cleavage, two missed cleavage sites, cysteine iodoacetyl acetylation (+57.021 Da) was set as a fixed modification, and glutamic acid and aspartic acid deamination (+0.98 Da) and methionine oxidation (+16 Da) were set as variable modifications. The relative molecular mass error of the parent ions was 15 ppm, and the isolation window was 4 Da. Results were set using software parameters: FDR <1%. Finally, all quantitative information was normalized, and significantly different proteins were screened according to |log2fold change| ≥ 0.26 and P-value <0.05. The pathways of the differential protein sets were annotated using IPA software.

### 2.11 PRM-based targeted proteomics (PRM-MS)

The protein samples from untargeted proteomics were analyzed using an Easy-Nalc1200 system (Thermo Fisher Scientific) combined with an Orbitrap Fusion Lumos mass spectrometer (Thermo Fisher Scientific). Peptides were separated with a C18 150 mm × 120 mm column (1.9 mm, 120 A˚, Dr. MaischGebH, Germany). Mobile phase A was a 0.1% formic acid aqueous solution, and mobile phase B was an 80% acetonitrile solution, with the gradient elution as follows: 0–8 min, 6%–12% B; 8–58 min, 12%–30% B; 58–70 min, 30%–40% B; 70–71 min, 40%–95% B; 71–78 min, 95%–95% B.

The collection mode was Full MS-PRM, the mass spectrometry spray voltage was 2.0 kV, and the ion transmission tube temperature was 320 °C. The RF Lens (%) was 50, the MS resolution was 60,000, and the scanning range was 350–2000 m/z. AGC was 1 × 105, the 4-bar isolation window was 1.6 m/z, the scanning range was 350–1800 m/z, the target MS/MS resolution was 30,000, and the cracking energy was 35 b y HCD cleavage. The target ions were specific peptides (Table 1) of the main proteins downstream of the PI3K/AKT signaling pathway.

**TABLE 1:** Unipeptide of proteins for PRM-based targeted proteomics.

Protein	Protein accession	Unique peptide
Pik3r1	P26450	FPAASSDNTEHLIK NESLAQYNPK SREYDRLYEEYTR QGC [+57]YAC [+57]SVVVDGEVK
Tgfbr3	O88393	VIAPDSIGFGK
AKT	P31750	MNDVAIVK VTMNEFEYLK

After data collection using the Data-dependent acquisition (DDA) method, the enzymatically cleaved peptides were identified qualitatively using Proteome Discoverer 2.4. By this, information such as retention time, parent ions, daughter ions used for quantification, and limit of quantification of the target peptides was determined. The target peptide ion database was built using Skyline software. Unipeptides containing target peptide sequences were chosen for PRM data collection and the established PRM method was utilized for the relative quantitative analysis of target proteins in samples from the aforementioned groups.

### 2.12 Statistical analysis

SPSS 23.0 software (SPSS Inc., IBM, United States) was used to analyze data from ELISA and PRM-based targeted proteomics, and GraphPad Prism 8.0 software (GraphPad Software Inc., CA, United States) was used for visualization. One-way ANOVA was used to assess differences among multiple groups and unpaired Student’s t-test for two groups when the data fit a normal distribution, and the data are presented as mean ± S.D.; for multiple comparisons, the Least Significant Difference (LSD) test (equal variance assumed) or the Games-Howell test (equal variance not assumed) were utilized. Non-normal data are presented as median and were analyzed using the non-parametric test Mann-Whitney U for two groups or Kruskal–Wallis for multiple groups. *P*-value < 0.05 was considered significant.

## 3 Results

### 3.1 Tracheal drip of bleomycin effectively induced CTD-ILD-like lung injury in mice

**FIGURE 1:**
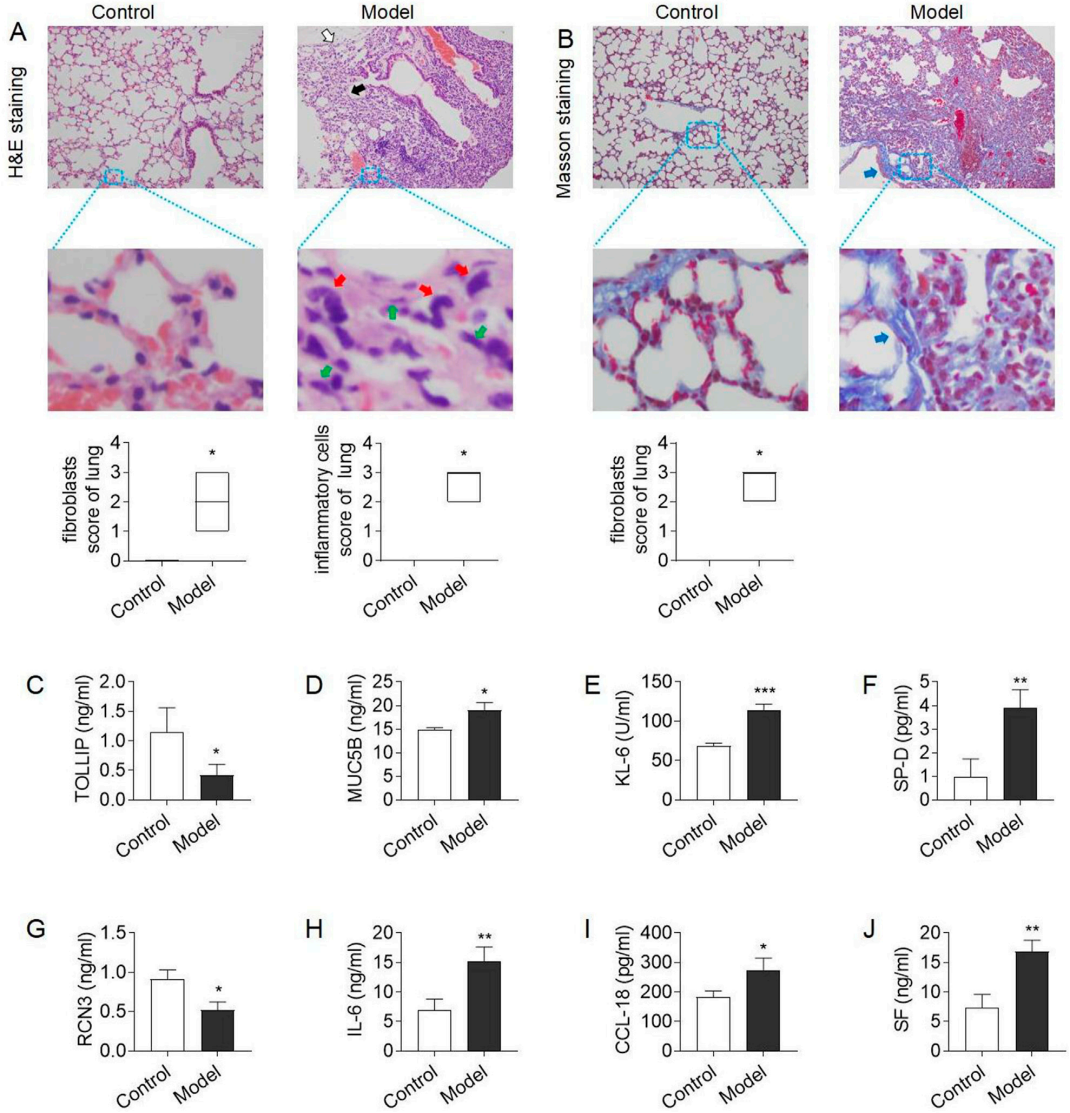
Model evaluation of CTD-ILD-like lung injury in mice **(A)** H&E staining of lung tissue (×200): mild to moderate atrophy of alveoli (white arrow), mild to moderate fibrous tissue proliferation in the interstitium and thickening of alveolar septa (black arrow), and increased fibroblasts (green arrow) and inflammatory cells (red arrow) in the model group, and alveolar septal thickening and interstitial inflammatory cell infiltration scores; **(B)** Masson staining of lung tissue (×200): a large amount of fibrillar collagen formation (blue arrows) in the model group, and fibrous hyperplasia score; **(C–D)** The level of serum TOLLIP and MUC5B, biomarkers of risk and predisposition in CTD-ILD **(E–G)** The level of serum KL-6, SP-D, and RCN3, biomarkers of epithelial cell dysfunction and extracellular matrix remodeling in CTD-ILD; **(H–J)** The level of immunoinflammation-related indicators IL-6, CCL18, and SF in serum. Data are presented as mean ± S.D. *P < 0.05, **P < 0.01, ***P < 0.001 for vs Control; ^#^ *P <* 0.05, ^##^ *P <* 0.01, ^###^ *P <* 0.001 for vs Model; Mann-Whitney U for figure **(A–B)**, and the Least Significant Difference test for figure **(C–J)**. *n* = 3 in figure **(A–J)**.

Bleomycin induced inflammations and fibrosis in the lung tissue of mice. As shown in the H&E-stained images (Figure 1A), mice in the model group had mild to moderate atrophy of alveoli, increased fibroblasts, mild to moderate fibrous tissue proliferation in the interstitium, significant thickening of alveolar septa and inflammatory cell infiltration. In contrast, the lung tissue structure was normal in mice of the normal control group. Masson staining results further indicated that the lungs of the model group exhibited fibrotic-like characteristics, with a substantial amount of fibrillar collagen formation, demonstrating significant pulmonary consolidation (Figure 1B). This suggests that bleomycin successfully induced lung tissue damage and fibrosis similar to CTD-ILD.

To further clarify the therapeutic role of PD + Cur in lung injury, it was subsequently tested for risk and predisposition biomarkers, epithelial cell dysfunction and extracellular matrix remodeling biomarkers, and immunoinflammation-related indicators for CTD-ILD, respectively.

It has been reported that overproduction of mucin 5 B (MUC5B) or a decrease in TOLLIP exacerbates lung injury and pulmonary fibrosis (Inoue et al., 2020; Li et al., 2020). And genetic variants in MUC5B and TOLLIP increase the risk or predisposition to ILD (Inoue et al., 2020; Li et al., 2020). The serum of bleomycin–treating model mice showed high expression of MUC5B (*P <* 0.05) and low expression of TOLLIP (*P* = 0.051) compared with the control group (Fig. C–D). This indicates abnormal changes in risk and predisposition biomarkers in CTD-ILD-like lung injury.

High expression of Kerb von denLungen-6 (KL-6) and lung surface-associated protein (SP-D) is closely related to epithelial cell dysfunction and extracellular matrix remodeling (Pan et al., 2025; Inoue et al., 2020; Stock et al., 2021), whereas Reticulocalbin 3 (RCN3) had a protective effect against this lesion (Jin et al., 2018). The results showed that the expression levels of serum KL-6 and SP-D were significantly higher (*P <* 0.01) and the expression level of RCN3 protein was lower (*P <* 0.05) in the model group mice compared with the control group (E-G). This suggests the presence of abnormally excessive immune inflammation in CTD-ILD-like lung injury.

C-C motif chemokine ligand 18 (CCL18) and IL-6 are key biomarkers of immunoinflammatory dysregulation and are involved in fibrotic processes (Phan et al., 2021; Zanatta et al., 2023). Serum ferritin (SF) is an iron storage protein that is highly expressed in inflammatory and autoimmune diseases (Ruscitti et al., 2017). As shown in Figure H–G, serum IL-6, CCL18, and SF expression levels were elevated in the model group *versus* the control group (*P <* 0.01, or *P <* 0.05). This showed that PD + Cur reduced the high expression of immunoinflammation-related indicators in CTD-ILD-like lung injury.

The above results demonstrate that bleomycin successfully induced lung injury resembling CTD-ILD in mice, characterized by immunoinflammation and fibrosis.

### 3.2 PD + Cur treatment effectively alleviated bleomycin-induced CTD-ILD-like lung injury

**FIGURE 2:**
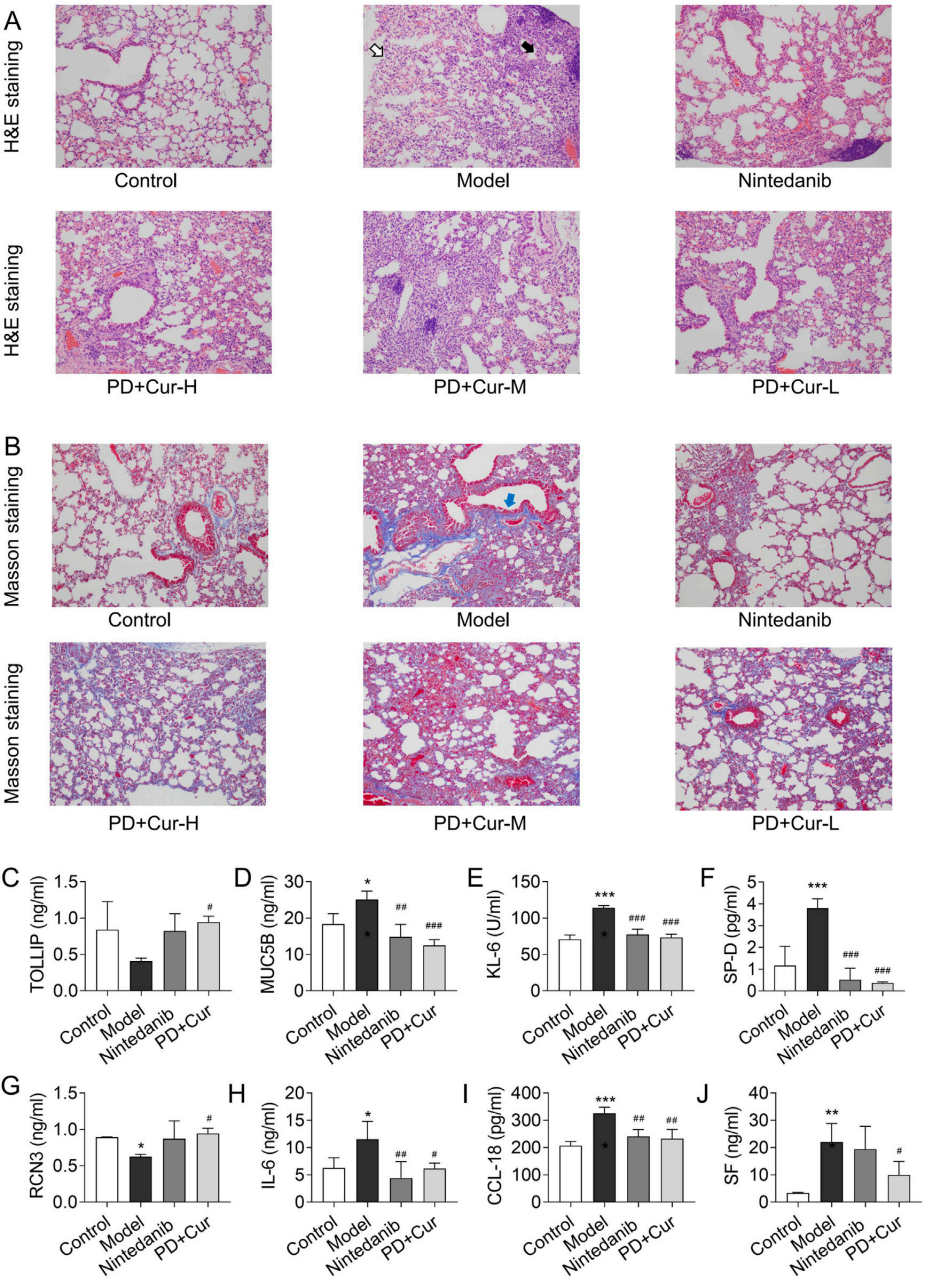
Efficacy of PD + Cur on CTD-ILD-like lung injury of model mice **(A)** H&E staining of lung tissue (×200): mild to moderate atrophy of alveoli (white arrow), and mild to moderate fibrous tissue proliferation in the interstitium and thickening of alveolar septa (black arrow) in the model group; **(B)** Masson staining of lung tissue (×200): a large amount of fibrillar collagen formation (blue arrows) in the model group; **(C–D)** The level of serum TOLLIP and MUC5B, biomarkers of risk and predisposition in CTD-ILD; **(E–G)** The level of serum KL-6, SP-D, and RCN3, biomarkers of epithelial cell dysfunction and extracellular matrix remodeling in CTD-ILD; **(H–J)** The level of immunoinflammation-related indicators IL-6, CCL18, and SF in serum. Data are presented as mean ± S.D. *P < 0.05, **P < 0.01, ***P < 0.001 for vs Control; ^#^ *P <* 0.05, ^##^ *P <* 0.01, ^###^ *P <* 0.001 for vs Model; Games-Howell for RCN3, the Least Significant Difference test for others. *n* = 3.

To assess the therapeutic effect of PD + Cur on bleomycin-induced CTD-ILD-like lung injury, H&E staining and Masson staining of lung tissues were performed. As shown in Figures 2A, B, alveolar lesions, thickened interstitium, and increased collagen fibers were improved in the bleomycin–induced model after treatment with PD + Cur and positive drugs, and the efficacy of PD + Cur at high, medium, and low doses was not significantly different.

To further evaluate the pharmacologic effects of PD + Cur, we tested risk and predisposition biomarkers, epithelial cell dysfunction and extracellular matrix remodeling biomarkers, and immunoinflammation-related indicators of CTD-ILD. Serum MUC5B was significantly reduced (*P <* 0.001) and TOLLIP was elevated (*P <* 0.05) in response to PD + Cur *versus* the model group (Fig. C–D). KL-6, SP-D, and RCN3 were all reversed in serum after intervention with PD + Cur compared to the model group (*P <* 0.001 or *P <* 0.05). And serum IL-6, CCL18, and SF were downregulated following treatment with PD + Cur compared with the model group (*P <* 0.01 or *P <* 0.05). This suggests that PD + Cur improved the biomarkers and immunoinflammatory factors associated with CTD-ILD.

The above results indicate that PD + Cur has good efficacy on lung tissue injury and fibrosis in bleomycin-induced CTD-ILD-like lung injury in mice, and the efficacy of PD + Cur at high, medium, and low doses was not significantly different.

### 3.3 PD + Cur modulated the PI3K/AKT/TGF-β pathway in CTD-ILD-like lung injury in model mice to ameliorate immune inflammation

**FIGURE 3:**
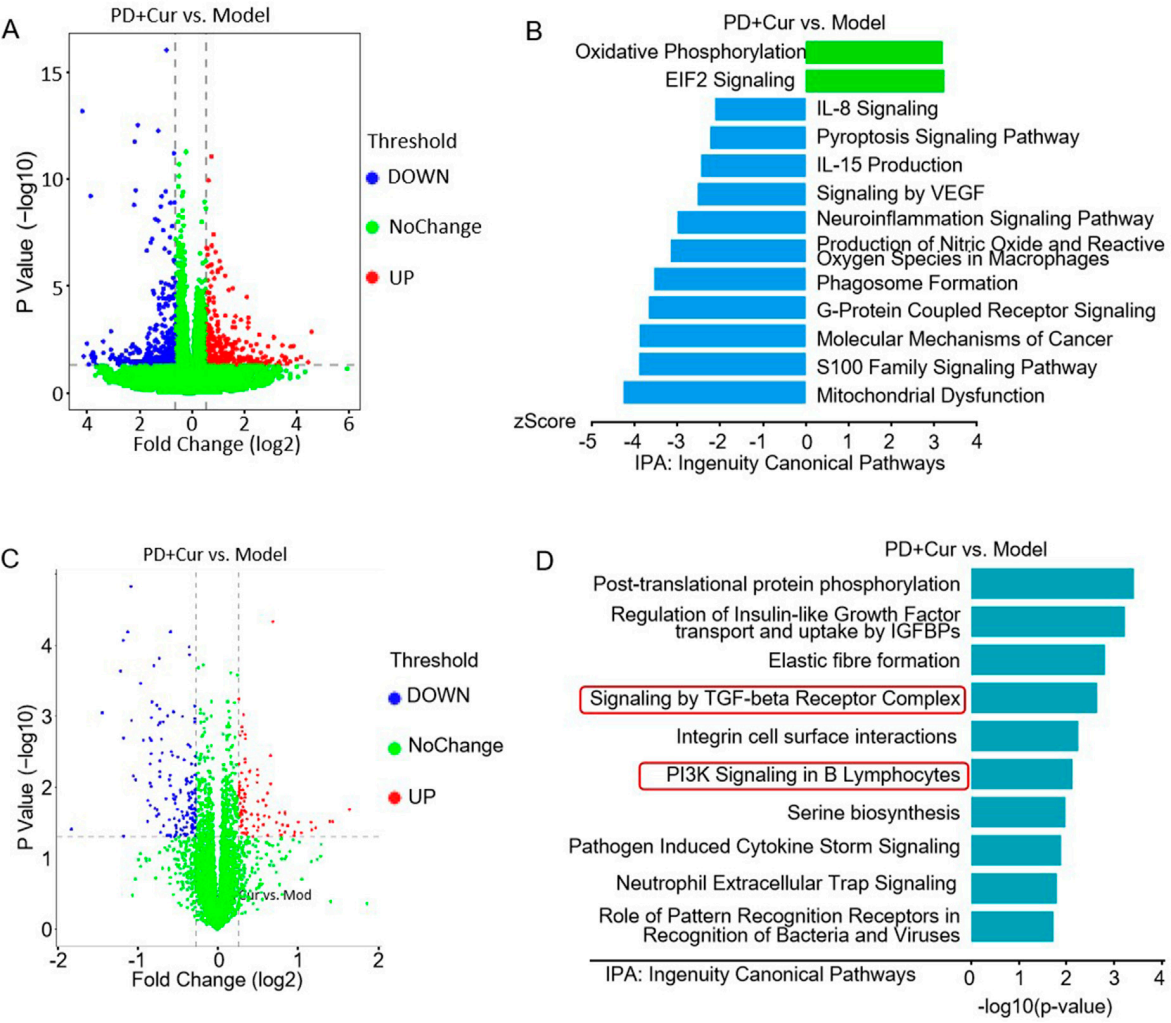
The results of RNA-seq and untargeted proteomics between PD + Cur vs Model **(A–B)** Differentially expressed genes (blue or red dots) and pathway enrichment between PD + Cur vs Model by RNA-seq analysis, *n* = 6; **(C–D)** Differentially expressed genes (blue or red dots) and pathway enrichment between PD + Cur vs. Model by untargeted proteomics. *n* = 6.

To explore the mechanism of PD + Cur for treating lung injury of CTD-ILD, we performed RNA-seq assays. The results of RNA-seq demonstrated that the development of CTD-ILD was associated with immune inflammation (Supplementary Figure S1), and PD + Cur modulates immunoinflammation-related signaling in CTD-ILD-like lung injury (Figures 3A, B), including IL-8 Signaling, IL-15 Production, Production of Nitric Oxide and Reactive Oxygen Species in Macrophages, Phagosome Formation, among others.

The untargeted proteomics further showed that the improvement of CTD-ILD-like lung injury by PD + Cur was associated with immunoinflammation (Figures 3C, D), such as PI3K signaling in B lymphocytes, production of nitric oxide and reactive oxygen species in macrophages, and signaling by TGF-beta receptor complex. Immunoinflammatory pathways involved in CTD-ILD-like lung injury also include PI3K signaling and TGF-β signaling (Supplementary Figure S2), which suggests that PD + Cur modulates the immune inflammation by regulating the PI3K/AKT/TGF-β pathway to improve the CTD-ILD-like lung injury of model mice.

### 3.4 Metabolomics suggested amino acid signaling plays an important role in PD + Cur’s regulation on CTD-ILD-like lung injury

We further performed metabolomics with combined transcriptomic and proteomic results to explore the targets of high-dose PD + Cur regulating immune inflammation in CTD-ILD-like lung injury in model mice.

**FIGURE 4:**
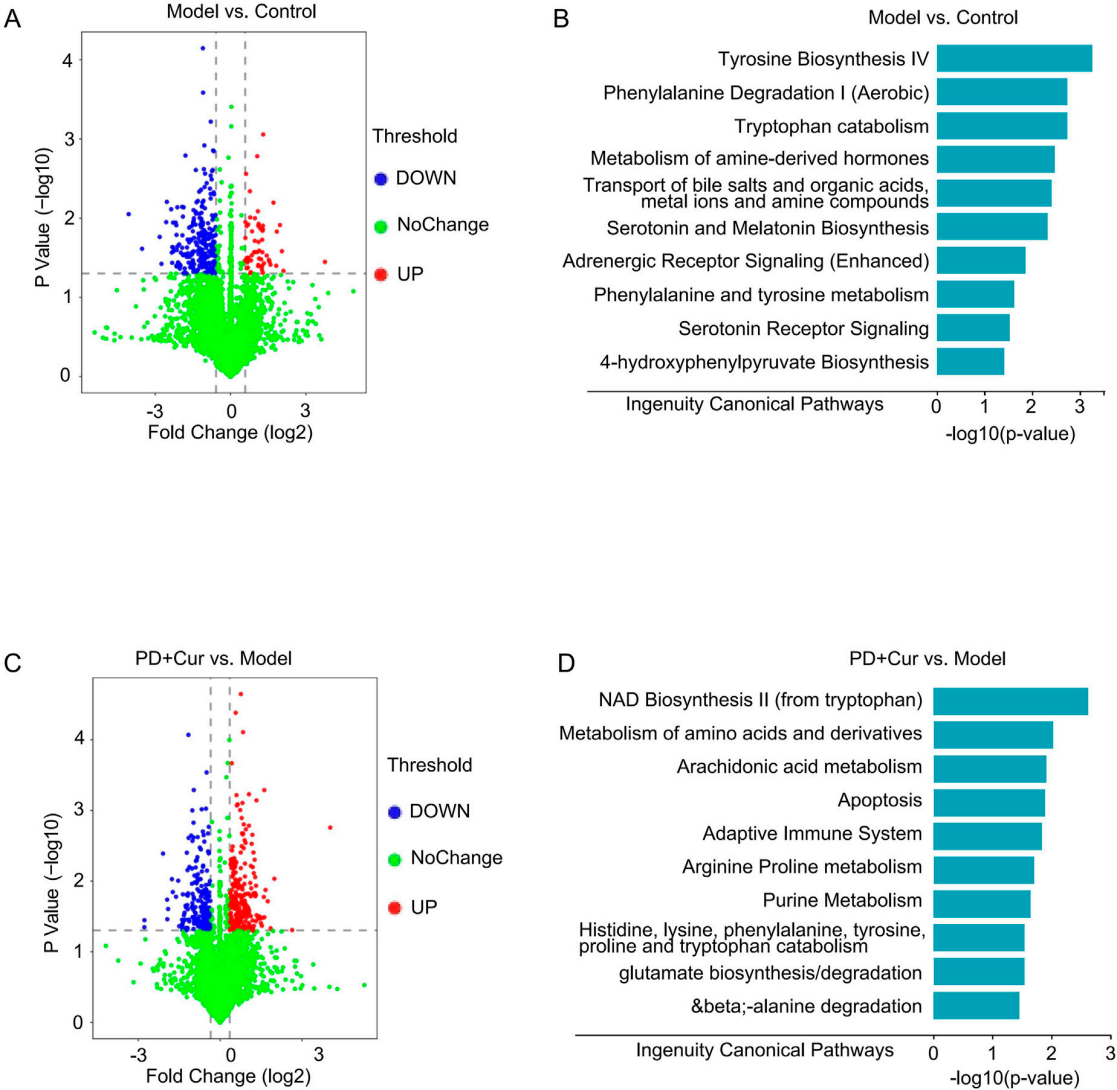
Metabolomics results of CTD-ILD-like lung injury of model mice with PD + Cur intervention **(A–B)** Differentially expressed genes (blue or red dots) and pathway enrichment between Model vs. Control; **(C–D)** Differentially expressed genes (blue or red dots) and pathway enrichment between PD + Cur vs. Model. *n* = 6.

Metabolomics analysis was performed by employing IPA software. The results showed (Figure 4) that metabolic pathways before and after modeling and before and after PD + Cur administration were closely related to amino acid metabolism, including tyrosine biosynthesis, phenylalanine degradation, tryptophan catabolism, metabolism of amine-derived hormones, phenylalanine and tyrosine metabolism, and so on. This suggests that PD + Cur may ameliorate CTD-ILD-like lung lesions by modulating amino acid-related pathways.

It was found in our previous study that PD + Cur attenuates immune inflammatory injury in rheumatoid arthritis by activating GABA receptors (Deng et al., 2021). According to studies, γ-aminobutyric acid (GABA) has a favorable mitigative effect on immune inflammation. GABA has been shown to inhibit lymphocyte- or macrophage-induced immune-inflammatory responses by activating its receptors in various immune-inflammatory diseases (Prud’homme et al., 2015). GABBR, the B-type receptor for GABA, can ameliorate inflammatory damage in autoimmune diseases such as rheumatoid arthritis, type 1 diabetes, and inflammatory bowel disease by modulating immune cells when activated (Prud’homme et al., 2015; Tian et al., 2021; Deng et al., 2023). In addition, GABBR was reported to act as an upstream protein to regulate the PI3K/AKT/TGF-β signaling pathway (Sun et al., 2020; Wang P et al., 2022). Therefore, it is hypothesized that PD + Cur may modulate downstream pathways by targeting GABBR and thus attenuating immunoinflammatory injury in the CTD-ILD-like lung injury of model mice.

### 3.5 PD + Cur regulates the PI3K/AKT/TGF-β pathway by targeting GABBR in CTD-ILD-like lung injury

To verify whether PD + Cur inhibits the PI3K/AKT/TGF-β pathway through targeted activation of GABBR to attenuate lung tissue injury in the bleomycin–induced model, the GABBR agonist baclofen was used to intervene in bleomycin–treating model mice and pharmacological and mechanistic studies were performed.

**FIGURE 5:**
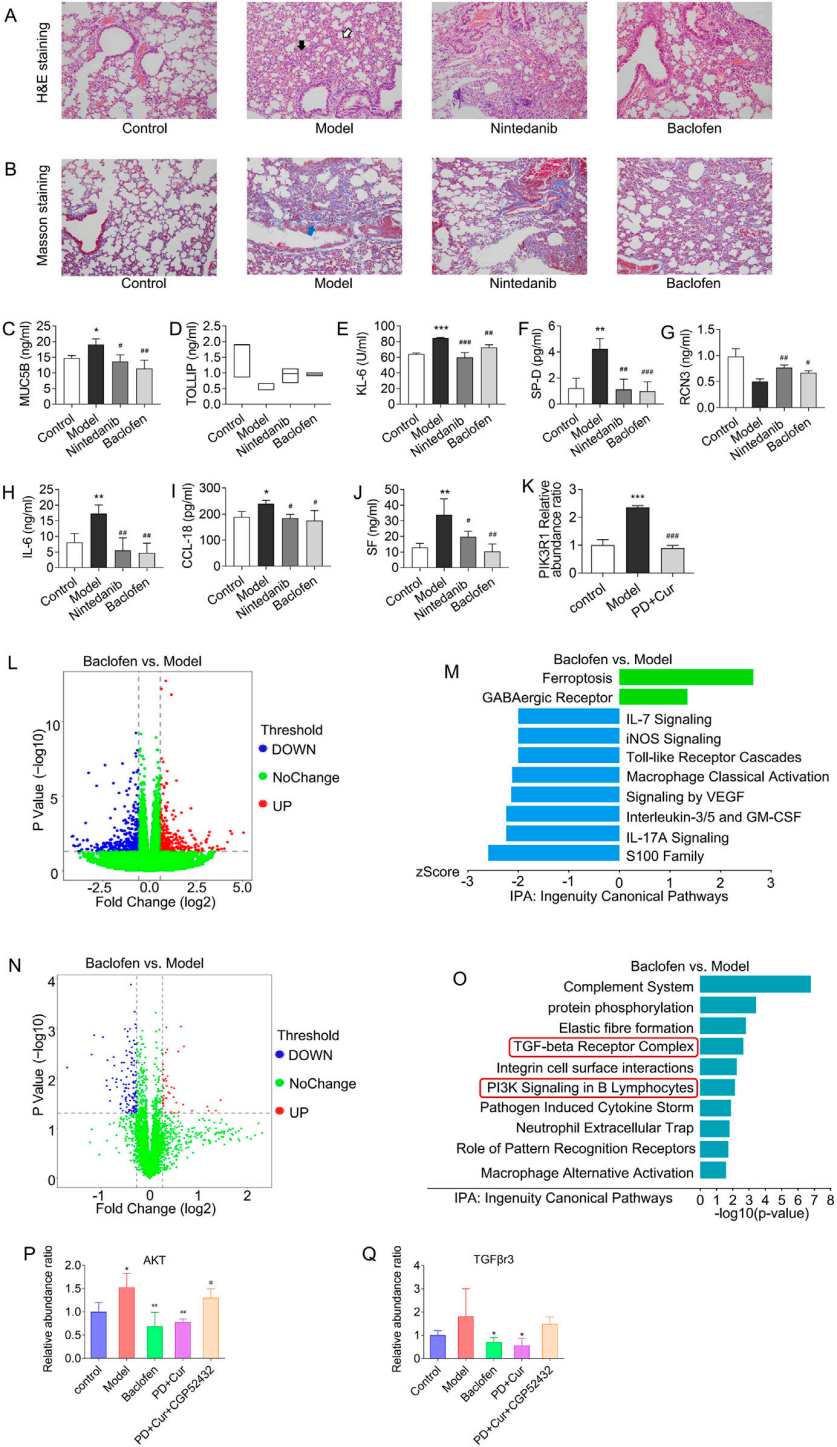
Alleviating immunoinflammatory injury in lung tissue of bleomycin–treating model by activating GABBR **(A)** H&E staining of lung tissue (×200): mild to moderate atrophy of alveoli (white arrow), and mild to moderate fibrous tissue proliferation in the interstitium and thickening of alveolar septa (black arrow) in the model group; **(B)** Masson staining of lung tissue (×200): a large amount of fibrillar collagen formation (blue arrows) in the model group; **(C–D)** The level of serum TOLLIP and MUC5B, biomarkers of risk and predisposition in CTD-ILD; **(E–G)** The level of serum KL-6, SP-D, and RCN3, biomarkers of epithelial cell dysfunction and extracellular matrix remodeling in CTD-ILD; **(H–J)** The level of immunoinflammation-related indicators IL-6, CCL18, and SF in serum **(K)** Relative expression levels of PI3KR1 in lung detected by PRM-based targeted proteomics; **(L–M)** Differentially expressed genes (blue or red dots) and pathway enrichment between Model vs. Control by RNA-seq analysis; **(N–O)** Differentially expressed genes (blue or red dots) and pathway enrichment between baclofen vs Model by untargeted proteomics; **(P–Q)** Relative expression levels of AKT and TGFβR3 in lung detected by PRM-based targeted proteomics. Data are presented as mean ± S. D in Fig. **(D–G)** and **(L–N)**, and as median in Fig. **(C)** *P < 0.05, **P < 0.01, ***P < 0.001 for vs Control; ^#^ *P <* 0.05, ^##^ *P <* 0.01, ^###^ *P <* 0.001 for vs Model; Kruskal–Wallis for TOLLIP, Games-Howell for RCN3, and the Least Significant Difference test for others. *n* = 6 for figure L–O, *n* = 3 for others.

The efficacy of GABBR agonists in CTD-ILD-like lung injury of model mice was first evaluated. The results showed (Figures 5A–J) that baclofen inhibited interstitial thickening and alveolar atrophy, decreased the expression of fibrillar collagen in lung tissues, and reversed the expression levels of the risk and predisposition biomarkers MUC5B, TOLLIP, and the epithelial cell dysfunction and extracellular matrix remodeling biomarkers KL-6, SP-D, and RCN3, and immunoinflammatory indicators IL-6, CCL-18, SF in the serum of model mice. These results suggest that GABBR agonist alone is also beneficial in alleviating lung tissue fibrosis of CTD-ILD-like lung injury.

The mechanism similarities between PD + Cur and baclofen were further analyzed to assess whether PD + Cur has GABBR agonist-like effects. RNA-seq results showed that baclofen mainly mediated immunoinflammation (Figures 5L, M), including IL-7 Signaling Pathway, iNOS Signaling, Macrophage Classical Activation Signaling, and Interleukin-3, and Interleukin-5 and GM-CSF signaling, *etc.* And untargeted proteomics showed that baclofen mainly regulates PI3K signaling and TGF-beta receptor complex signaling. We then performed an untargeted proteomic for PI3-kinase subunit p85-alpha (PI3KR1 - the activation subunit of PI3K), which showed that PI3KR1 was significantly upregulated in lung tissues of the bleomycin–treating model (*P <* 0.001) compared to the control group and significantly downregulated after intervention with PD + Cur or baclofen (P < 0.001) (Figure 5K). The above results suggest that the effect mechanism of baclofen on CTD-ILD-like lung injury is highly consistent with that of PD + Cur as described previously.

In addition, we further validated the pathway mechanism of PD + Cur by intervening in the CTD-ILD-like lung injury of model mice with baclofen and GABBR inhibitor CGP52432, respectively. And used targeted proteomics to detect key proteins downstream of PI3K. Serine/threonine-protein kinase (AKT) can be directly activated by PI3K to initiate downstream signaling. Transforming growth factor beta receptor type 3 (TGFβr3) binds to TGF-β and is involved in the capture and retention of TGF-β for signaling presentation. As shown in Figures 5P–R, the protein expression level of AKT, and TGFβR3 was increased in the lung tissues of the model mice compared with the control group, which were decreased after the intervention of PD + Cur and Baclofen. In contrast, the downregulation of PIK3R1 downstream proteins AKT and TGFβr3 by PD + Cur was inhibited by the addition of CGP52432 (i.g.) in the PD + Cur group.

## 4 Discussion

### 4.1 Inflammation leads to pulmonary fibrosis in CTD-ILD, and PD + Cur may improve pulmonary fibrosis in CTD-ILD by suppressing inflammation

It is a well-established fact that inflammation is an important trigger of regeneration and fibrosis (Mack, 2018). In many diseases, inflammation often manifests first, and over time, inflammatory lesions can undergo a fibrotic transformation, such as inflammatory bowel disease (D’Alessio et al., 2022), chronic hepatitis (Koyama and Brenner, 2017), and chronic kidney disease (Wu et al., 2022). This fibrotic process is achieved by crosstalk between inflammation and fibroblasts in the diseased tissue. In the alveoli, inflammation stimulates the activation of alveolar fibroblasts, which induces fibrotic lesions in the lungs (Savin et al., 2022; Tsukui et al., 2024). In lung injury of CTD-ILD, excessive inflammatory cascade response is an important factor in promoting proliferation and activation of lung fibroblasts and continued progression of the lung lesions (Cerro Chiang and Parimon, 2023). According to the study, in the bleomycin-induced pulmonary fibrosis model, the same inflammatory response in the lungs was initiated, followed by a gradual increase in fibrosis, resulting in a predominantly fibrotic lesion with coexisting inflammation as a feature of lung injury (Schniering et al., 2019). The same lung injury characteristics were observed in the model induced by bleomycin in our study, whereas PD + Cur ameliorated lung inflammation and inhibited the progression of fibrosis in the model mice through inhibition of the inflammatory pathway PI3K/AKT. Thus, our findings suggest that the therapeutic efficacy of PD + Cur on pulmonary fibrosis in CTD-ILD is achieved by inhibiting inflammation.

### 4.2 The multi-omics results suggest that the GABBR/PI3K/AKT/TGF-β pathway is the mechanism by which PD + Cur ameliorates CTD-ILD-like lung injury

The transcriptomic and proteomic enrichment pathway results suggested that CTD-ILD-like lung injury was mainly related to immunoinflammation and fibrosis, which coincided with the pathological changes in CTD-ILD lungs reported in the literature. The results also implied that PD + Cur could modulate the immune-inflammatory and fibrotic pathways, so it was targeted to explore how PD + Cur could ameliorate the inflammation and fibrosis in lung tissues. The regulatory pathways of PD + Cur on immune inflammation involve Pi3K Signaling in B Lymphocytes, IL-8 Signaling, IL-15 Production, S100 Family Signaling Pathway, Production of Nitric Oxide and Reactive Oxygen Species in Macrophages, *etc.* It has been reported in the literature that the PI3K pathway is located downstream of IL-8 (Meng et al., 2023; Wu T et al., 2024), IL15 (Shenoy et al., 2014), and S100 (Geng et al., 2023; Otake et al., 2024), thereby mediating macrophage polarization and activation of immune inflammation (Zhao et al., 2020). In addition, regulatory pathways by PD + Cur also include signaling by TGF-beta receptor complex, signaling by VEGF, and elastic fibre pathway, which are associated with fibrotic lesions (Adamo et al., 2021; Amano et al., 2021). PI3K and TGF-β have been reported to regulate the fibrotic process (Peng et al., 2022; Wang J et al., 2022). This suggests that the PI3K/TGF-β pathway may be the central mechanism by which PD + Cur ameliorates lung fibrosis in CTD-ILD.

Metabolomics results (Figure 4) suggest that the effects of PD + Cur are related to amino acid. Our previous study showed that PD + Cur attenuates immune-inflammatory damage in rheumatoid arthritis by activating GABA receptors but not other amino acid receptors (Deng et al., 2021). GABA has been shown to inhibit lymphocyte- or macrophage-induced immune-inflammatory responses by activating its receptors in various immune-inflammatory diseases (Prud’homme et al., 2015). GABBR, the B-type receptor for GABA, was reported to act as an upstream protein to regulate the PI3K/AKT/TGF-β signaling pathway (Sun et al., 2020; Wang P et al., 2022). Although metabolomics results were also enriched for other amino acid pathways, including tyrosine biosynthesis, phenylalanine degradation, tryptophan catabolism, metabolism of amine-derived hormones, phenylalanine and tyrosine metabolism, and so on. However, more literature argues that GABA-related pathways regulate fibrosis and immune inflammation (Deng et al., 2023; Prud’homme et al., 2015; Sun et al., 2020; Tian et al., 2021; Wang P et al., 2022). Therefore, it is hypothesized that PD + Cur may modulate downstream pathways by targeting GABBR and thus attenuating immunoinflammatory injury in the model mice.

We further validated this by intervening in model mice with Baclofen, an agonist of GABAB. The pathway enrichment results of the transcriptome and proteome (Figure 5) indicated that Baclofen coincidentally regulated Pi3K Signaling in B Lymphocytes, interleukin-related pathway, TGF-beta receptor complex, signaling by VEGF, and elastic fibre pathway, but not Oxidative Phosphorylation, EIF2 signaling, and other pathways. This provides strong evidence that GABBR/PI3K/AKT/TGF-β is most likely the key mechanism by which PD-Cur ameliorates lung injury in CTD-ILD, rather than the other pathways in Figures 3–5. Therefore, we will next discuss in detail the mechanism of action of PD-Cur on immunoinflammatory injury and fibrosis in lung tissue of CTD-ILD with respect to the GABBR/PI3K/AKT/TGF-β pathway.

### 4.3 GABBR may be a key target of PD + Cur to inhibit PI3K/AKT inflammatory signaling

It has been suggested that insufficient GABA levels and its receptor activity may be an important factor in pulmonary fibrosis diseases and autoimmune diseases. Overexpression of GABA receptors has been reported to reduce fibronectin expression in idiopathic pulmonary fibrosis, whereas knockdown of GABA receptors promotes fibrosis (Zhang et al., 2022). GABA and its receptor agonists ameliorate immunoinflammatory damage in rheumatoid arthritis by inhibiting the p38 MAPK pathway (Deng et al., 2021; Kelley et al., 2008). In this study, we demonstrated that PD + Cur has GABA agonist-like effects by GABBR agonists and inhibitors, thereby modulating the immune inflammatory response and lung fibrosis in CTD-ILD-like lung injury model mice.

GABA can inhibit the immune-inflammatory response caused by abnormal lymphocyte and macrophage activation through activation of its receptors (Prud’homme et al., 2015), and has been shown to be used in a variety of immune-inflammatory diseases, such as rheumatoid arthritis, type 1 diabetes mellitus, and inflammatory bowel disease (Prud’homme et al., 2015; Tian et al., 2021; Deng et al., 2023). PI3K/AKT signaling has been reported to be an important downstream pathway in the regulation of immune inflammation by GABBR (Sun et al., 2020; Wang et al., 2022). Pathway enrichment by proteomics showed that the mechanism of action of PD + Cur is closely related to PI3K signaling in B lymphocytes and macrophage activation signaling pathways. In rheumatoid arthritis and systemic sclerosis, there is an imbalance in B-lymphocyte differentiation, which is characterized by excessive activation of pro-inflammatory effector B cells (Beffs) producing autoAbs and reduced levels of anti-inflammatory regulatory B cells (Bregs), leading to excessive inflammatory response (Guo et al., 2015; Hu et al., 2023). For B lymphocytes, their development, survival, activation, and differentiation are regulated by the PI3K/AKT signaling pathway (Ramadani et al., 2010; Zhu et al., 2024). B lymphocyte proliferation cycle arrest or apoptosis can be induced by inhibiting the PI3K/AKT pathway (Lou et al., 2024; Wu C et al., 2024). In addition, cells of the immune system have been reported to produce GABA and express its receptor GABAB (Zhang et al., 2021). The upregulation of the PI3K/AKT pathway was reversed by PD + Cur and baclofen in lung tissues of CTD-ILD-like lung injury model mice in the present study, while CGP52432 could inhibit the effect of PD + Cur. Therefore, it can be speculated that PD + Cur can inhibit the overproliferation of Beffs to reduce immune inflammation by targeting GABBR to suppress the PI3K/AKT pathway.

### 4.4 PD + Cur may inhibit TGF-β-mediated fibrosis by inhibiting the PI3K/AKT pathway

The PI3K/AKT pathway is likewise a driver of M2 macrophage differentiation (Wang et al., 2021). For macrophages, the M1 type plays a role in inflammatory damage in CTD-ILD-prone diseases such as rheumatoid arthritis and desiccation syndrome (Chen et al., 2024; Yuan et al., 2024). However, M2 macrophages have been reported to be overdifferentiated in these diseases as well (Gong et al., 2024; Pan et al., 2023; Soldano et al., 2024). Although M2 macrophages are necessary for the initiation of tissue repair procedures, their continued activation or recruitment may lead to fibrosis through over-secretion of TGF-β (Gong et al., 2024; Wynn and Vannella, 2016). TGF-β is an important inducer of fibrosis, and its type 3 receptor, TGFβR3, was significantly upregulated in the lung tissue of the model mice in this study and was reversed by PD + Cur. Thus, PD + Cur may alleviate TGF-β pathway-mediated fibrous tissue proliferation in CTD-ILD-like lung injury model mice by regulating GABAB/PI3K/AKT to inhibit M2 overdifferentiation. Some other studies have argued that GABA promotes macrophage activation toward M2, which may be due to the bi-directional modulation of its differentiation by GABA in different pathological states, which needs to be further researched.

**FIGURE 6:**
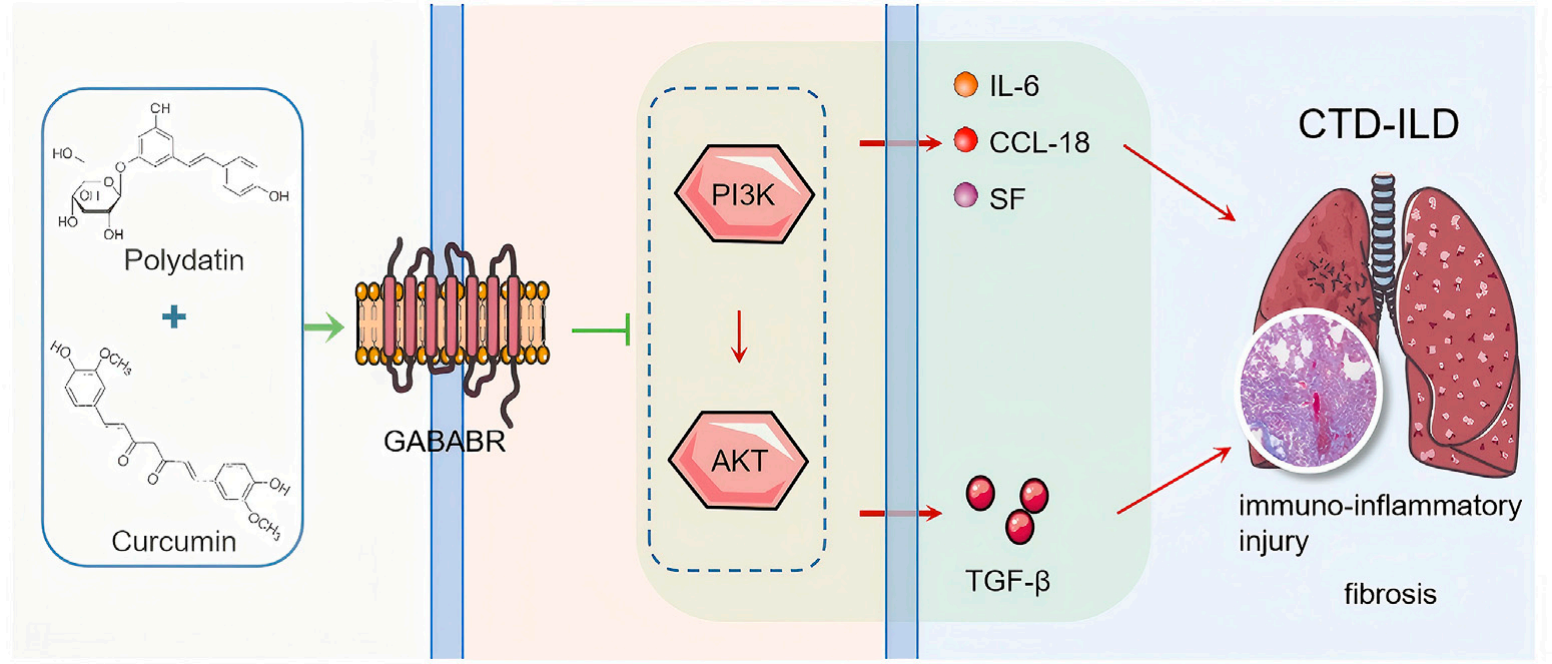
PD + Cur inhibits the PI3K/AKT/TGF-β pathway by targeting and activating GABBR.

In summary, we preliminarily conclude that PD + Cur has an therapeutic effect on lung tissue lesions in bleomycin-induced CTD-ILD-like lung injury model mice. PD + Cur inhibits the PI3K/AKT/TGF-β pathway by targeting and activating GABBR (Figure 6), which reduces the excessive immune-inflammatory response and fibrosis in the lung tissues of CTD-ILD.

## 5 Shortcomings and prospects

There are some limitations to this study. Our findings suggest that the ameliorative effect of PD + Cur on pulmonary fibrosis in CTD-ILD is achieved. Regrettably, while we validated that PD-Cur modulates GABBR and thus PI3K/AKT/TGF-β for therapeutic effects through GABBR agonists, we did not further validate that PI3K/AKT/TGF-β is the only downstream pathway or the most highly correlated downstream pathway for PD-Cur to modulate GABBR in this disease. These remain to be further explored in follow-up. Additionally, if the role of the GABBR/PI3K/AKT/TGF-β pathway in CTD-ILD could be validated in clinical samples, it would significantly enhance the clinical relevance of our findings. However, due to the limited availability of well-characterized biospecimens (particularly lung tissues) from CTD-ILD patients, as well as the complex ethical and regulatory approvals required for clinical sample collection, we are currently unable to perform these critical clinical validations. In future work, we will pursue these validations through ongoing collaborations with clinical centers.

## Data Availability

The original contributions presented in the study are publicly available. This data can be found in the National Center for Biotechnology Information (NCBI) repository (accession number: PRJNA1268941).
