# Supplementary material for: Polydatin-curcumin formulation alleviates CTD-ILD-like lung injury in mice via GABBR/PI3K/AKT/TGF-β pathway
Source: Front Pharmacol. 2025 Jun 5;16:1573525. doi: 10.3389/fphar.2025.1573525 (PMC12176778; doi:10.3389/fphar.2025.1573525)
Supplement: Supplementary file 1 [file Supplementaryfile1.docx]

Supplementary Material

**Supplementary Figure 1**. The results of RNA-seq between Model vs Control

(A) Differentially expressed genes (blue or red dots); (B) pathway enrichment of differentially expressed genes. n=6.

**Supplementary Figure 2**. The results of untargeted proteomics between Model vs Control

(A) Differentially expressed genes (blue or red dots); (B) pathway enrichment of differentially expressed genes. n=6.

**Supplementary Figure 3**. The levels of c-FOS, c-JUN, EGR1, COX2, CCL4, and IKBKG

The expression levels of c-FOS, c-JUN, EGR1 and CCL4 based on transcriptome sequencing but not detected by proteomics; The expression levels of COX2 and IKBKG based on proteomics.

**
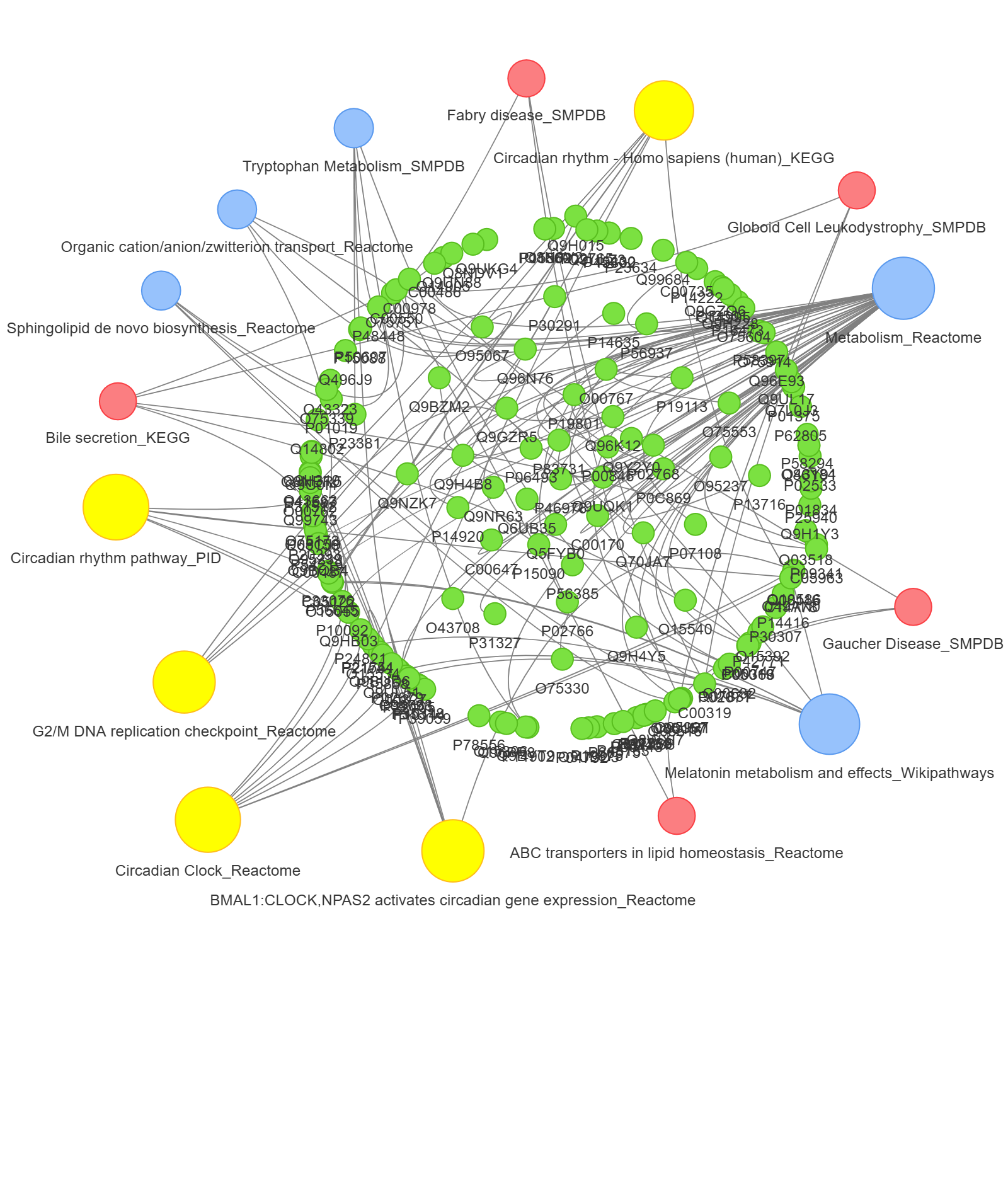
**

**Supplementary Figure 4.** Integrated Transcriptome sequencing and Metabolomics Analysis

Proteomics+Metabolomics (blue), Proteomics (yellow), Metabolomics (red), and Objects (green)


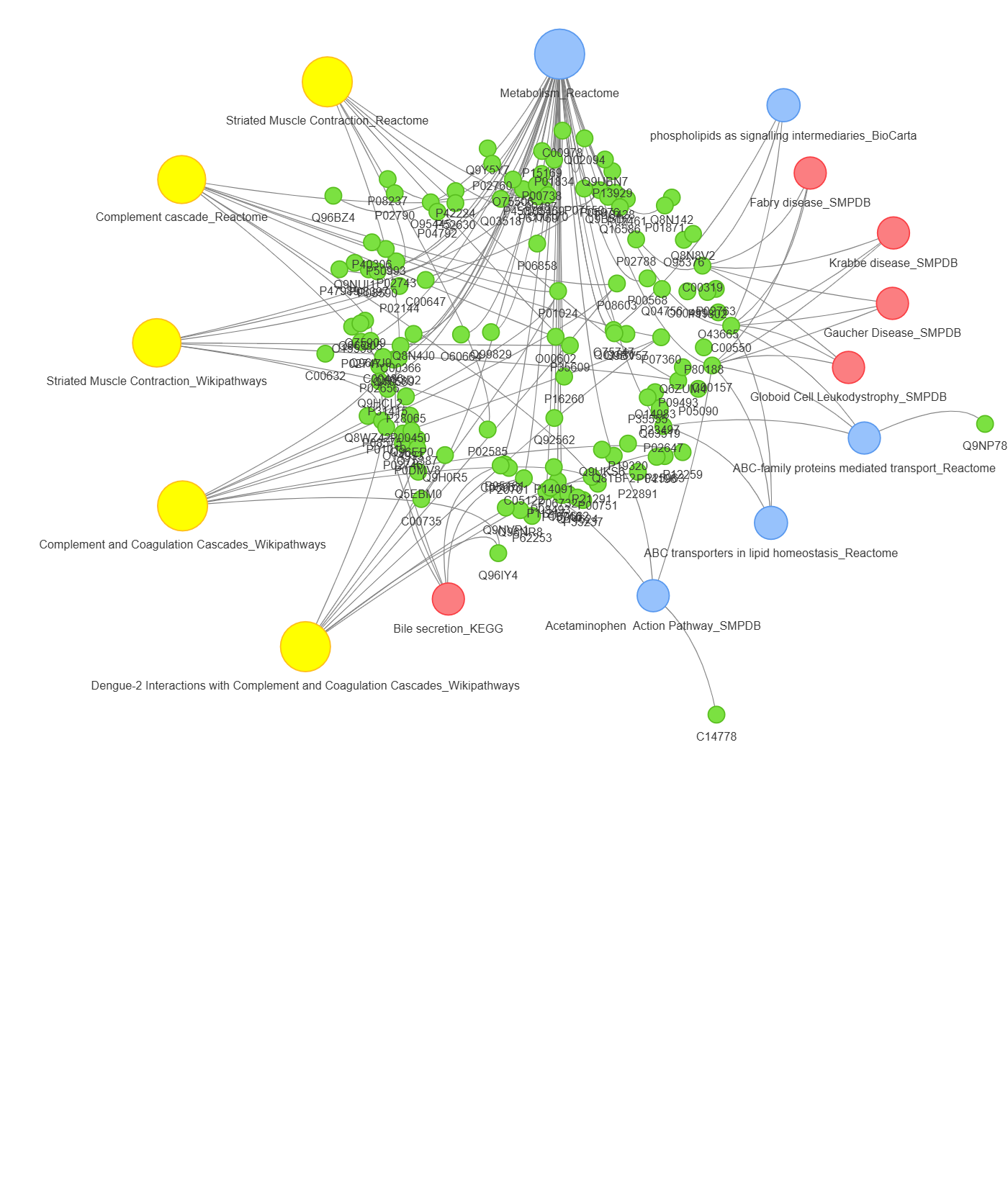


**Supplementary Figure 5**. Integrated Proteomics and Metabolomics Analysis

Proteomics+Metabolomics (blue), Proteomics (yellow), Metabolomics (red), and Objects (green)

# Pharmacokinetic evaluation of PD+Cur

The absorption and distribution of a drug significantly influence its pharmacological activity and duration of action. To elucidate the in vivo metabolic behavior of PD and Cur, we investigated their absorption and distribution profiles following intraperitoneal (i.p.) administration in rats. Male Sprague-Dawley rats were administered 7.46 mg/kg of PD+Cur via i.p. injection. Plasma and tissue samples (heart, liver, spleen, lung, and kidney) were collected at 10 min, 30 min, 1 h, and 3 h post-administration. The concentrations of PD and Cur were quantified using LC-MS/MS.

The plasma concentration-time curves of PD and Cur were plotted (Supplementary Figure 3A-B), and key pharmacokinetic parameters were derived. Both compounds reached peak plasma concentrations (Cmax) within 10 min after i.p. injection. However, their elimination profiles differed significantly. PD exhibited rapid clearance, with most of the compound eliminated within 2 h (short half-life). Cur maintained detectable plasma levels for up to 6 h, indicating a longer half-life and sustained systemic exposure.

Both PD and Cur were widely distributed across all examined tissues (Supplementary Figure 3C-F). The rank order of tissue concentrations at each time point was as follows: PD showed highest concentrations in lung > liver > spleen > heart > kidney at 10 min, kidney > liver > lung > spleen > heart at 30 min, kidney > spleen > lung > liver > heart at 90 min, and spleen > liver > lung > kidney > heart at 180 min; while Cur consistently distributed as spleen > liver > heart > lung > kidney at 10 min, spleen > liver > lung > kidney > heart at 30 min, spleen > liver > lung > kidney > heart at 90 min, and spleen > liver > lung > kidney > heart at 180 min.

These findings provide critical insights into the in vivo behavior of PD+Cur, supporting its pharmacological effects. The rapid absorption and tissue-penetrating properties of PD, combined with the prolonged circulation of Cur, suggest complementary pharmacokinetic profiles that may contribute to their combined therapeutic efficacy.

**Supplementary Figure 6**. Pharmacokinetic evaluation of PD+Cur

(A-B) The plasma concentration-time curves of PD/Cur; (C-F) Time-course distribution profile of PD/Cur in heart, liver, spleen, lung, and kidney tissue.
